# Rabies in Uganda: rabies knowledge, attitude and practice and molecular characterization of circulating virus strains

**DOI:** 10.1186/s12879-020-4934-y

**Published:** 2020-03-06

**Authors:** Michael Omodo, Meriadeg Ar Gouilh, Frank Norbert Mwiine, Anna Rose Ademun Okurut, Noelina Nantima, Alice Namatovu, Maria Flavia Nakanjako, Emmanuel Isingoma, Eugene Arinaitwe, Martin Esau, Simon Kyazze, Milton Bahati, Franklin Mayanja, Patrick Bagonza, Richard Akule Urri, Mary Nanfuka Lovincer, Esther Nabatta, Eugene Kidega, Chrisostom Ayebazibwe, Gladys Nakanjako, Joseph Sserugga, Deo Birungi Ndumu, Robert Mwebe, Kenneth Mugabi, Jean-Paul Gonzalez, Musa Sekamatte

**Affiliations:** 1 Ministry of Agriculture Animal Industry and Fisheries, National Animal Disease Diagnostics and Epidemiology Centre, P.O. Box 513, Entebbe, Uganda; 2 Normandy University, EA2656, GRAM2.0 - Groupe de Recherche sur l’Adaptation Microbienne, UNICAEN – UNIROUEN, Caen University, 14000 Caen, France; 3 Virology Department, University Hospital Center of Caen, 14000 Caen, France; 4 College of Veterinary Medicine, Animal resources and Biosecurity, Makerere University, Kampala, Uganda; 5 Ministry of Health, National One Health Platform: Zoonotic Disease Coordination Office, Entebbe, Uganda; 6 Georgetown university school of medicine, Washington, DC USA

**Keywords:** Rabies, *Lyssavirus*, Molecular-epidemiology, KAP, Phylogeography

## Abstract

**Background:**

Rabies is a deadly preventable viral disease that affects all warm-blooded animals and widespread in many regions including Africa. The disease remains of major public health importance in Uganda.

The purpose of this study was to establish Knowledge, Attitude, Practice (KAP) of Rabies in Moyo and Ntoroko districts and to characterize Rabies virus (RABV) strains from seven districts of Uganda with consistent prevalence of rabies.

**Methods:**

KAP survey data were collected based on animal biting history by interviewing the head of the veterinary departments, the medical centers and selected households from the study sites. Data were obtained from 84 households in Ntoroko and Moyo districts. Thirty-five (35) brain samples were collected from bovine, dogs, goats, foxes, jackals ad sheep between 2011 and 2013. Samples were tested using fluorescent antibody test (FAT), One step RT-PCR (following RNA extraction) and partial RABV N gene was sequenced by Sanger method before phylogenetic and phylogeographic analyses of sequences.

**Results:**

Scarcity of post-exposure prophylaxis services in the health centers was noted. Poor attitude of wound washing and deficiency of knowledge on how to handle wounds related to dog bites and the significance among household participants lacked. There is a high risk of rabies infection due to a limited dog’s vaccination. Dog biting episodes in humans were of 75.00 and 62.50% in Moyo and Ntoroko districts respectively. Twenty-seven (27) samples tested positive for rabies by FAT and PCR. Ugandan sequences were closely related (97% nucleotide id) to the rabies virus sequences from Tanzania, Rwanda, Burundi, Nigeria, Central African Republic and Sudan with both the “Africa 1A” and “Africa 1B” RABV clades represented. A putative new clade 1D was also detected.

**Conclusions:**

Rabies remains a public health hazard in Uganda. There is urgent need to establish advocacy programs in both schools and communities to curtail the spread of rabies. Increasing the knowledge regarding wound washing, post-exposure prophylaxis and dogs vaccination would enhance prevention of rabies. A strong collaboration between medical and veterinary sectors under a one health platform is required to ensure sufficient preventative services to the communities.

## Background

Unearthing globally the lyssaviruses remains of persistent scientific interest and importance to both public and animal health. Indeed, lyssaviruses are recognized to cause fatal encephalitis, generally referred to as rabies infection [1]. The RABV is a neurotropic agent of the *Lyssavirus* genus, *Rhabdoviridae* family. RABV is responsible of Rabies (i.e. disease in human and animals) and it is the only viral pathogen agent associated with 100% fatality in the absence of treatment and after the clinical manifestation of the disease [2]. Human get infected by direct contact (i.e. through biting, scratching or licking of wounded skin) with RABV infected animals including dogs, raccoons, skunks, bats, jackals and foxes. Dogs account for more than 99% of the human cases and consequently Rabies is worldwide recognized as a high-priority zoonotic disease.

RABV present an RNA genome that encodes five highly conserved genes including: the nucleoprotein (N), Phosphoprotein (P), matrix protein (M), glycoprotein (G), and a viral RNA polymerase (L). The virus nucleoprotein (N) plays significant role in replication and transcription and has been widely used for molecular typing, and phylogenetic studies of the virus [3–5].

In Africa, molecular detection of RABVusing the N-gene clustered the isolates with China lineage 2 that co-circulate with Africa lineages in Monrovia and Liberia where the first case in Africa was reported [5]. RABV strains from domestic dogs and livestock in Africa are divided into 4 main lineages including: Africa 1A, with a broad distribution across the continent and predominant in northern and eastern Africa; Africa 1B found mainly from eastern and southern Africa; Africa 2, from western Africa; and, Africa-4 from western Africa [6].

Rabies is endemic in Uganda and significantly affects public health and livelihoods with an average of 16,414 human dog bites registered annually [7].

At National Animal Disease Diagnostics and Epidemiology Centre (NADDEC), Ministry of Agriculture, rabies diagnosis is performed following the gold standard of virus detection test using the Fluorescent Antibody Test (FAT), as recommended by WHO and OIE [8] . When brain specimens from both domestic and wild animals are tested at NADDEC for RABV antigen detection, results are timely reported to district veterinary officers responsible for controlling the spread of the disease. Although, Uganda spends an average of UGX 7 billion ($1.9 m) on rabies management, mainly for procurement of pre- and post-exposure prophylaxis (PEP) vaccines in humans and rabies vaccination for dogs and cats, effective control of rabies is still a challenge. The true burden of the disease is not yet established due to underreporting. Therefore, rabies in Uganda remains a neglected disease and one of the seven priority zoonotic diseases listed by Uganda ministry of Health and Agriculture.

The present study aims at investigating Knowledge, Attitude, Practice (KAP) of rabies among selected households of the Moyo and Ntoroko Districts and to characterize the RABV strains circulating among several districts of Uganda and to estimate their contribution to the global diversity of rabies in Africa.

## Methods

### Study sites and material

**Fig. 1:**
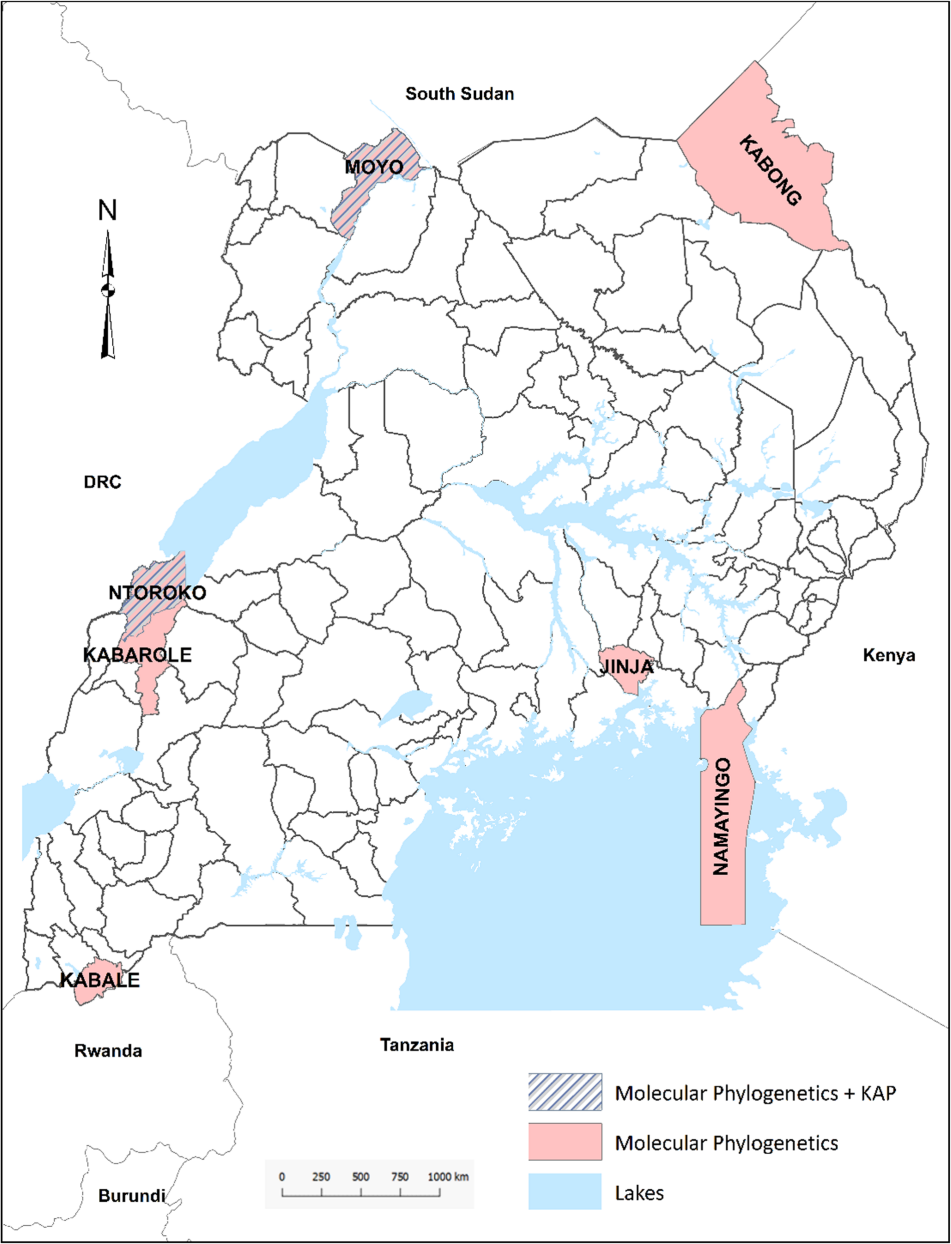
Rabies study sites (Districts), Uganda (2009–2012). Stripped Blue = District with cross-sectional study and brain tissue analyzed for virus detection; Light red = District that provide only brain tissue for virus detection. Map adapted from https://geojson-maps.ash.ms. Geo-coordinates of localities: 03°39′N 31°43′E, 01°06′N30°24′E, 00°25′24″N 33°12′14″E, 01°15′S 30°0′E, 03°32′24″N 34°07′30″E, 00°17′N 33°51′E, 00°17′N 33°51′E

A total of 35 brain tissues were collected from suspected dogs, cattle, goats, sheep, fox, jackals in seven districts of Uganda (Moyo, Ntoroko, Jinja, Kabaale, Kabongo, Namayingo and Kabarole districts) (Fig. 1).

### Data collection from selected households

The survey was conducted in the two districts of Moyo and Ntoroko to collect data based on dog biting history reported by the selected households. These two districts were selected for the KAP study because of their high reporting of rabies suspected samples to NADDEC, for laboratory rabies confirmation, as compared to any other districts in Uganda from 2009 and 2013. Eighty-four (Moyo, *n* = 44; Ntoroko, *n* = 40) households affected by dog bites participated in the study, as well as the local government health centers and medical private centers where first aid treatment due to dog bites were offered. Data was collected using a well-designed tool with both objective and open-ended questions (Supplementary Material: SM0 - questionnaire). Collected parameters include knowledge status of the households about rabies, handling of dog bites by local community, age, dog bite site, death associated with suspected rabies and, affected livestock. Dogs were defined as owned or confined, free roaming or stray dogs in the community.

### Retrospective review of registers in rural medical settings

An animal bite was defined as any injury or wound inflicted by a suspected rabid animal to humans and registered during the review period. In 2013, patient and availability of anti-rabies vaccine, PEP protocols in the selected health facilities were reviewed. Data on age, gender, number of patients bitten by suspected rabid dogs and other animal species involved in biting people were also recorded. The survey also focused on challenges in the management of dog bite victims in the visited medical centers.

### Retrospective review of registers from district veterinary department

From 2009 to 2013, the access to the livestock disease register provided the number of dogs vaccinated, target areas, suspected number of rabid animals, animal species involved in biting humans and health status of animals. Methods of controlling stray dogs if any were recorded as well as the number of livestock, wildlife and dogs suspected of rabies, all data provided by the district veterinary officer.

### Data management

Data were registered using Microsoft Excel 2007 (Microsoft, Seattle, WA, USA), and analyzed using STATA 10 package for statistical analysis [9]. The Fisher’s exact test or the Chi-square X^2^ test was applied according to the number of observations with a statistically significant threshold set at a *P* < 0.05 (95% confidence interval).

A number of categorical variables were stratified (2 by 2 tables) according to the district of origin in order to assess the significance among the tested variables of interest in Moyo and Ntoroko. Differences in frequency of observations between districts and the association between key variables of interest using uni-variable analysis were calculated.

The statistical analysis performed was descriptive, qualitative and quantitative among the 84 households of Moyo and Ntoroko districts. The data captured included: age, gender, knowledge about rabies (transmission, clinical signs and management of dog bites), vaccination and treatment, ownership, care and number of bites. The reason for using the non-probabilistic method of sampling was to explore the knowledge, attitude and practice about rabies in the specifically affected households.

### Samples and rabies virus detection

The brain samples were collected from dead animals which where owned by private individuals. Some were from farmers and fishermen on along river Nile. Some were from dogs that were straying in these communities. The affected goats, cattle, and sheep brain samples used in this study were willingly provided by farmers for laboratory testing, after suspected clinical signs of rabies disease were observed. Verbal consent to collect samples of dead Jackals that were found lying dead in Kidepo National Game park was given by wildlife authority, because in Uganda only National Animal Disease Diagnostics and Epidemiology Centre (NADDEC) has the capacity to diagnose rabies using the direct fluorescent antibody test method. Thirty-five (35) brain tissue samples sourced from dogs, cattle, goats, sheep, foxes and jackals were tested at NADDEC for RABV antigen detection using Fluorescent Antibody Test (FAT) as previously described by OIE in 2013 [8]. Brain specimen were stored in 50% glycerol-saline at − 80°c and later shipped to Instituto Zooprofilattico Sperimentale delle Venezie (IZSVe, Italia) for RABV culture and viral RNA extraction, and one-step RT-PCR and sequencing.

### Viral RNA extraction

Under class II Biosafety cabinet, 30 mg of each sample was homogenized with motor and pistol to mechanically lyse the nerve cells, then 3.5 μL of cell suspension on RAI PBS, guanidinium and ß-mercaptoethanol were lysed using a vortex mixer, followed by one-minute centrifugation (12,000). The entire procedure of RNA extraction was performed by using the Nucleospin RNA II kit, following the manufacturer’s instructions (Macherey–Nagel GmbH & Co., Düren, Germany). One hundred μl of supernatant was pipetted and placed in new sterile RNA collection tubes with columns to be washed with buffer and centrifuged 1 min (12,000 rpm). After DNA digestion on silicate membrane and centrifugation, RNA was eluted from the membrane. The purified RNA was eluted in a final volume of 40 μl and subjected for amplification by One Step RT-PCR.

### One-step RT-PCR (reverse transcription- polymerization chain reaction)

**Fig. 2:**
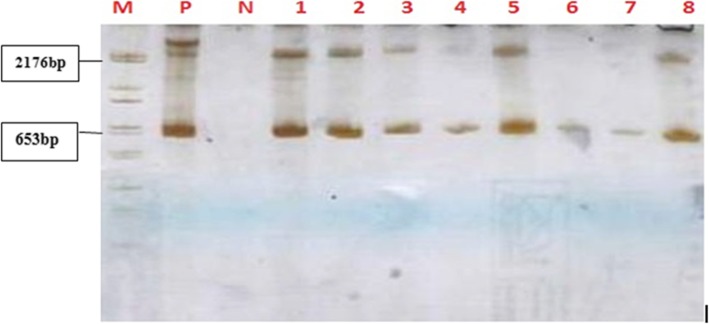
Acrylamide gel electrophoresis of RT-PCR products targeting the Rabies virus N-gene of positive Rabies samples. Lane M = Roche DNA marker; Lane P = positive control; Lane N = negative control; Lanes 1 to 8 are selected positive brain samples showing a 653 bp band size. A 7% acrylamide gel electrophoresis showing representative results obtained with primers targeting the N-gene of Rabies virus

RT-PCR was performed using the One-Step RT-PCR kit (Qiagen®, Germany), following the manufacturer’s instructions. Published primers were used to amplify a 603 bp region of the N gene as previously described [10]. This region of the N gene is highly conserved and involved in the nucleoprotein region (450–451 amino acids) that protects the genome from nucleases and mediates evasion or induction of type 1 interferon. PCR products were visualised on 7% acrylamide gel electrophoresis (Fig. 2).

### Nucleotide sequencing and analysis

PCR products were purified with ExoSAP-IT (USB Corporation, Cleveland, OH) and sequenced in both directions using the Big Dye Terminator v3.1 cycle sequencing kit (Applied Biosystems, Foster City, CA), the Performa DTR Ultra 96-well kit (Edge Biosystems, Gaithersburg, MD) and a 16-capillary ABI PRISM 3130xl Genetic Analyzer (Applied Biosystems, Foster City, CA, USA). Nucleotide sequences were then assembled using SeqScape v.2.5 program (Applied Biosystems 78,744 Austin, Tx).

### Phylogenetic and Phylogeographic analyses

Phylogenetic analysis was based on partial N gene sequences. Original sequences were trimmed and aligned with a set of sequences representing the genetic diversity of RABV using MAFFT [11]. First phylogenetic analyses were performed in maximum likelihood using PhyML, implemented in Seaview [12]. Then, Bayesian phylogenetic analyses were implemented in BEAST (version 1.8.4) [13], using the most appropriate model according to the corrected Akaike Information Criterion (AICc) from Jmodeltest2 [14]. The model included the general time reversible model of substitution with a gamma distribution and a proportion of invariant sites (GTR + I + G). The random starting tree used the coalescent (constant size) model. A relaxed molecular clock with an uncorrelated lognormal distribution was implemented in combination with a tip date sampling [15]. The MCMC (Markov-Chain Monte Carlo) was launched 3 times with 10 E7 iterations and 10 E3 samples each in order to reach Effective Sampling Size (ESS) values above 200 after logs and trees files were combined using log Combiner with a 10% discard. In order to evaluate spacial diffusion dynamics of rabies in Africa, a discreet geographic model (i.e. based on country and associated biogeographic zone) were coded as discrete traits for each genetic sequence and incorporated into the model as an additional data partition. Symmetric substitution model and a social network using BSSVS (Bayesian Stochastic Search Variable Selection) procedure were used to infer a discrete ancestral state reconstruction and to estimate most probable geographical diffusion pathways [16]. This discrete model was complemented by a model in continuous space, by using geographic coordinates of taxa with Brownian random walk used as continuous trait model with bivariate trait representing latitude and longitude values. The ancestral state reconstruction (geographic coordinates of nodes) was performed in continuous space and for each ancestor to evaluate the most probable diffusion route of lineages. The maximum clade credibility tree with continuous traits was then combined with GeoJSON map file (https://geojson-maps.ash.ms) to produce the graphical output using spreaD3 software version 0.9.7 [16].

## Results

### Knowledge, attitudes and practices of selected households on rabies

In this study both Ntoroko and Moyo districts interviewed population equally presented their dogs for vaccination against rabies. In Moyo 38.64% of respondents owned dogs as compared to 57.50% in Ntoroko. Among the interviewed households in Moyo and in Ntoroko, 45.45 and 47.50% had access to veterinary services, respectively. Also, 36.36 and 42.50% of the respondents who owned dogs presented their dogs for parental vaccination against rabies in Moyo and Ntoroko respectively. The world rabies day (WRD) events participation represented 13.64 and 2.5% of respondents in Moyo and Ntoroko, respectively.

The association between knowing rabies (presentation, risk, transmission) and knowledge of WRD showed that all respondents had some knowledge about rabies while 10% only truly knew about rabies disease. Among the respondents who took their dogs for vaccination, 20.00% had some knowledge about rabies and 40.51% were knowledgeable about rabies. In both districts over 90% all respondents were aware about rabies as a disease of dogs, however only 18.99% of the responds washed their wounds before seeking medical treatment to nearest regional hospital.

The most affected age range of humans with dog bites episodes was between 11 and 20 years and female accounted for most cases with 51,35% and 63,89 in Moyo and Ntoroko, respectively (Table 1).

**Table 1:** Age distribution of rabies human cases bitten by animals in the households of Moyo and Ntoroko districts, Uganda (2012–2014)

Age	Moyo	Ntoroko
< 5^a^	4/37 (10.8)^b^	4/36(11.11%)
6–10	2/37 (5.41%)	12/36 (33.33%)
11–20	18/37 (48.65%)	13/36(36.11%)
21–30	3/37 (8.11%)	3/36 (8.33%)
> 35	10/37(27.03%)	4/36 (11.11%)
Total	37	36

Legend: ^a^ = year old; ^b^Positive / total tested (%)

The study revealed that respondents in the two districts of Ntoroko and Moyo were knowledgeable about rabies and in agreement (*P* = 0.665). From these 84 respondents in Ntoroko and Moyo districts, majority of the interviewed households knew the clinical signs and how the virus is transmitted through dog bite (Table 2). In Moyo and Ntoroko, respectively 63 and 55% of the dog-bite victims that visited the hospital, were not aware of wound cleansing and report to either hospital or to the village health centres without cleaning the wound but visited the health units for PEP.

**Table 2:** Knowledge, attitude and practices on rabies of different households of Ntoroko and Moyo districts, Uganda (2009–2012)

*Observation*		*Moyo*	*Ntoroko*	P**
Knowledge	Rabies	42/44 (95.45)*	37/42 (92.50)	0.665
	World Rabies Day	8/ 445 (18.18)	2/40 (5.00)	0.092
Mode of rabies transmission	Water	0/44 (0)	1/40 (2.50)	0.476
	Bites	41/44 (93.2)	37/42 (92.5)	1.000
*Dog’s Clinical signs*	Salivation	31/44 (70.5)	29/42 (72.5)	1.000
	Biting objects	p//44 (52.27)	29/40 (72.50)	0.057
	Eating	0/44 (0)	1/40 (2.5)	0.477
	Sleeping	1/44 (6.27)	3/40 (7.5)	0.345
	Excitement	5/44 (11.36)	3/40 (42.50)	0.001
Management (*Vaccine)* after bites	Dogs	16/44 (36.36)	17/40 (42.50)	0.580
	Cats	3/44 (6.82)	3/40 (7.50)	1.000
Treatment	Wash wounds	8/44 (18.18)	2/40 (17)	0.935
	PEP	28/44 (63.64)	22/40 (55)	0.421

Legend: * = Positive / total tested (%); ** = *p* value at95% confidence limit

Only 36.36 and 42.50% took their dogs to the community vaccination centres in Moyo and in Ntoroko, respectively. Farmers where not attentive presenting their dogs for vaccination even some of them reported to be bitten by stray dogs. In several instances, stray dogs were then killed by shooting, chemical or poisoning and others infected stray dogs died from rabid disease.

In Moyo only 25.00% young individuals attempted to answer the questionnaires, while in Ntoroko there were 30%. In the adult age-class 43.00% respondents were from Moyo and 37.50% from Ntoroko. In the highest age-class, 27.27% respondents were from Moyo and 27.50% from Ntoroko district. In Moyo, 70.45% of respondents were male and 62.50% in Ntoroko district, while 29.55 and 37.50% were female, respectively.

### Rabies virus detection and genetic analysis

**Fig. 3:**
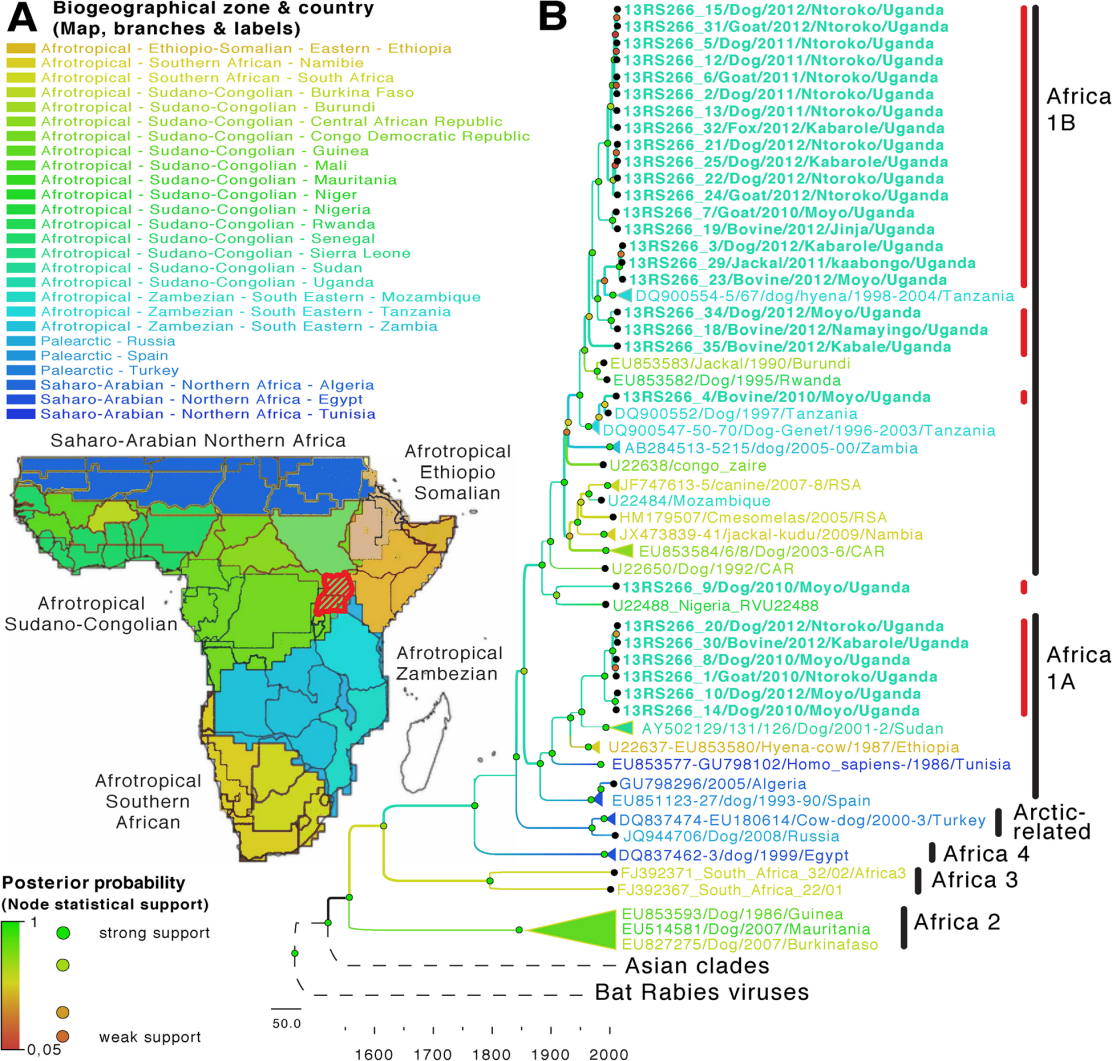
Biogeography of rabies in Africa. **a** Biogeographical zones (including biogeographica data from [17] with kind permission of the authors) and countries of Rabies dispersion in sub-Saharan Africa; **b** locations of probable ancestral nodes inferred from the Bayesian time-resolved phylogeny of 84 rabies virus including 27 virus strains isolated in Uganda and the other major strains circulating in Africa. Biogeographical zones and countries are colored and reported on taxa labels and branches (ancestral state reconstruction). Nodes are colored according to robustness (posterior probability): red for weak and green for strong (significant) support. The five main biogeographical zones are summarized from [17]. The localization of Uganda and original sequences from this study are highlighted in red on the map and in the tree, respectively. The probable dates of divergence are given by the time scale depicted below the tree. Many groups have been collapsed to enhance readability of this figure (expended tree and maximum likelihood phylogeny with 222 taxa available in supplementary material, SM1 and 2)

**Fig. 4:**
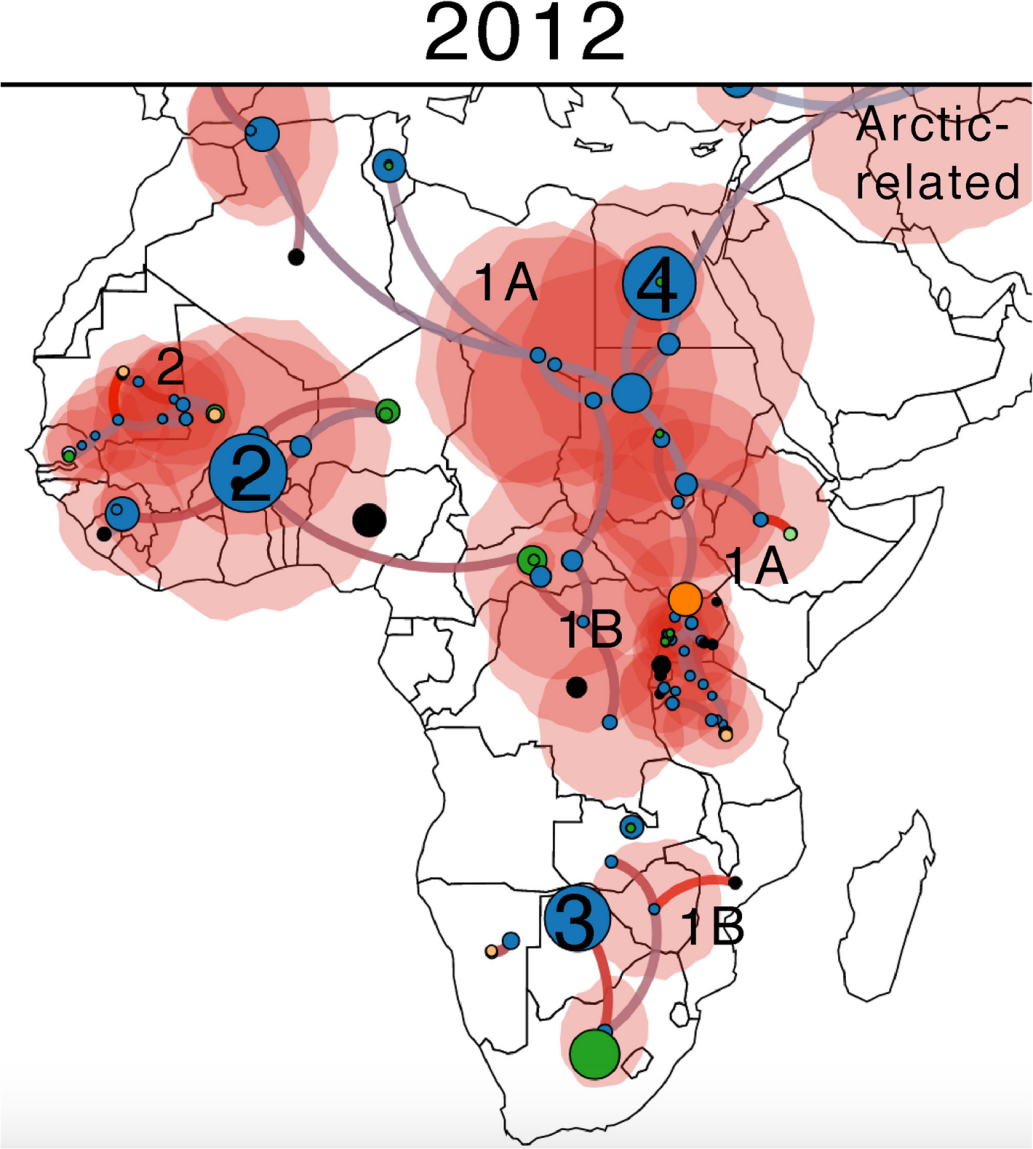
Phylogeographic diffusion of rabies in Africa according to a continuous model. Spatial-temporal phylogenetic reconstruction using the same genetic dataset as for Fig. 3: 84 rabies virus including 27 virus strains isolated in Uganda and the other major strains circulating in Africa. Nodes and terminal taxa are represented by circles. Blue nodes are internal probable nodes, with position resulting from ancestral reconstruction state. Other (colored) nodes represent terminal taxa. Main lineages Africa 1 to 4 are represented by number 1 to 4, respectively. Node size represents the median length of the corresponding branch (and here reflects the age of the node). Red polygons represent the uncertainty range around internal node position (location 80% HPD). Branches are color gradient depicted according to their rate of evolution (2, 34E-4 [1,29E-4 – 3,31E-4] 95%HPD; blue: slower rate; red: faster rate). Maps were downloaded from https://geojson-maps.ash.ms

Among thirty-five brain samples tested, 77.1% were positive for rabies by FAT (Table 3), and were unequally distributed among the different species (Table 4). The phylogenetic analyses showed that the RABVs detected in the study belonged to the cosmopolitan lineage, with both Africa 1A and Africa 1B clades detected (Figs. 1, 2, 3). Four distinct sub-clades (sub-lineages) of RABV circulating contemporaneously in Uganda were identified (Fig. 3b). Four sub-clades belong to the African 1B lineage and another (6 sequences) to the African 1A group (Fig. 3). In the present study, no Africa 1C related virus (SM2) was detected. According to the discrete and continuous geographical models developed (Figs. 3, 4, SM1, 2 and 3), the rabies genetic clustering correlates to biogeography (Fig. 3a) and the RABV detected in Uganda are related to several phylogenetic lineages coming from several African biogeographical zones (Figs. 3 and 4). Isolates identified as 13RS266_29/Jackal/2011/Kabongo/Uganda, 13RS266_3/Dog/2012/Kabarole/Uganda, 13RS266_23/Bovine/2012/Moyo/Uganda clustered with Tanzanian carnivore isolates (DQ900555/Dog/2001/Tanzania, DQ900567/spotted_hyena/2004/Tanzania, DQ900554/Dog/1998/Tanzania) which belongs to Africa 1B lineage circulating in East and Southern Africa.

**Table 3:** Source of the brain specimen tested for rabies by Fluorescent antibody test from seven districts of the four regions of Uganda (Central, Western, Eastern, and Northern) (2009–2012)

District of origin	Number of specimen /positive (%)
*Moyo*	11 / 8 (72.7%)
*Ntoroko*	13 / 12 (92.3%)
*Jinja*	1 / 1 (100%)
*Namayingo*	1 / 1 (100%)
*Kabongo*	3 / 1 (33.3%)
*Kabarole*	5 / 3 (60%)
*Kabale*	1 / 1 (100%)
Total	35 / 27 (77.1%)

**Table 4:** Animal species samples tested by direct Fluorescent Antibody Test for Rabies antigen

Species	Total tested	Positive
*Dog*	19	15
*Cattle*	7	6
*Goats*	5	4
*fox*	1	1
*Jackals*	3	2
Total	35	27 (77.1%)

A dog isolate from Moyo district (13RS266_9/Dog/2010/Moyo/Uganda), strongly clustered with a Nigerian strain (U22488_Nigeria_RVU22488) (Fig. 3 and SM1). Together they form a clade that clearly diverges from the root of all other Africa 1B.

Six Ugandan virus isolates from Northern and South Western Uganda (13RS266_10/Dog/2012/Moyo/Uganda, 13RS266_14/Dog/2010/Moyo/Uganda, 13RS266_20/Dog/Ntoroko/Uganda, 13RS266_1/Goat/2010/Ntoroko/Uganda, 13RS266_30/Bovine/2012/Kabarole/Uganda, 13RS266_8/Dog/2010/Moyo/Uganda) were close to Sudanese isolates from dogs (AY502129/Dog/2001/Sudan, AY502131/Dog/2002/Sudan, AY502126/Dog/2001/Sudan) belonging to the lineage Africa 1A circulating in dogs from Northern and eastern Africa (Fig. 3 and SM1).

Virus strains of the African 1B group detected in Uganda (mostly from Moyo, Ntoroko and Kabarole districts, 2010 to 2012), clustered as a sister-clade of RABV that circulated in Sudan since 2001 and appear to share a common ancestor among the Afrotropical-Ethiopia-Somalian Biogeographical zone (i.e. Ethiopian clade) (Fig. 3). The relaxed molecular clock analysis suggests that the diversification of this clade occurred during the second half of the twentieth century. Phylogenetic analyses link the circulation of one lineage 1B in Uganda (detected from 2010 to 2012 in all localities sampled) to Tanzanian viruses and the episodic presence of a second lineage (in 2010 in Moyo), also to a Tanzanian lineage.

## Discussion

Rabies disease is endemic and remains an important public health challenge in Uganda, because dog rabies is poorly controlled and transmission of the rabies virus to healthy humans or animals occurs mainly through bites of infected dogs.

Children, mainly under 20 years old, account for over 50 % (50%) of the dog bite cases, with females being the most impacted. This study highlights factors that contribute to knowledge, attitudes and practices on handling rabies outbreaks in the rural communities of Uganda. Such risk factors should be targeted to reduce transmission and human death due to rabies. Apart from collection of quantitative and qualitative data from the study districts, the study captured circulating rabies virus genotypes from livestock, dogs, Jackals and foxes.

Both circulating rabies virus genotypes Africa 1A and B in wild canines and dogs were detected using brain samples after molecular characterization in the laboratory.

According to the study, participants lacked comprehensive knowledge about the disease. They were ignorant about the significance of wound washing and reporting. They believed that only domestic dogs and foxes transmit rabies to humans. According to the study, vaccination of rabies was covered in high risk areas only, therefore to effectively control the transmission and spread of rabies, it’s necessary to conduct vaccination exercise on a wider coverage. Since Uganda and Tanzania are neighbouring countries, they have similar rural settings and structures. Such findings are in agreement with a KAP study conducted in Tanzania by [18].

Though most of respondents knew rabies and its transmission pathways through dog bites, they lacked knowledge regarding risk factors and prevention. Factors influencing transmission and spread of rabies in these areas under study include limited knowledge, inappropriate practices, poverty and ignorance hence implying higher risk of developing rabies in such communities. Therefore, Intensive and impactful awareness interventions are needed to address a number of negative factors contributing to rabies infection and death among the local communities.

In the Ntoroko and Moyo districts forty (40%) interviewed population owned a dog. Less than Fifty percent (< 50%) of the respondents in both study districts never submit their dogs for vaccination against rabies. Although, dogs were the most affected domestic species confirming their critical role in rabies epidemiology [19], rabid suspected goats were reported to have bitten people in both Ntoroko and Moyo (unpublished) districts. Eighty (80%) of sampled goats involved in biting both humans and other animals and eighty six (86%) of tested cattle, were confirmed positive for RABV. Likewise, the disease was previously reported by wildlife authorities in Kidepo National valley park where twenty eight (28) jackals were found dead and confirmed positive for RABV antigen using FAT at National Animal Disease Diagnostics and Epidemiology Centre [20].

Most of the dog-bitten victims traversed long journeys to seek PEP services in the district referral hospital. Health centers provided only first aid treatment of wounds washing with clean water, soap and disinfectants such as iodine. In some private clinics, the PEP services were available but costly as previously observed [21]. Only 1/5 hospitals in the west Nile region provided PEP services.

While dog bites in human are a serious public health burden [22], in our study, over 70% of respondents who go to clinic did not wash wounds before seeking medical treatment. This is crucial because this simple measure decreases the risk to develop infection [23]. In agreement with a previous study [24], 40% of people of Ntoroko and Moyo districts are children victims of dog bites and that can be attributed to their playful behaviour with dogs. Both households had basic knowledge about rabies, but most of the respondents had no prior knowledge about rabies clinical sign in infected animals while all believed that only dog is responsible of rabies transmission. In addition, with respect to all variables studied, there was no significant difference between Moyo and Ntoroko districts. The majority of respondents declares to not wash the wounds (70%), indicating a lack of basic prevention practices knowledge in affected populations. Indeed, between the two districts, 1/10th only of the respondents had knowledge about “World Rabies Day”, however none of them participated. While more than 90% had no rabies knowledge, 20% of the interviewed households had knowledge about dog vaccination but less than half of them presented their dogs for vaccination.

Phylogenetic analyses compared 27 RABV sequences obtained from dogs, goats, cattle, jackals and fox from seven districts of Uganda to other regional strains available from the literature and Genebank. The phylogenetic analyses suggest a diversified circulation of RABV Africa 1 clade (cosmopolitan) in Africa echoed in our Uganda sampling where we found four distinct sub-groups of RABV Africa 1 (Fig. 3, SM1 and 2). Therefore, Africa 1A and Africa 1B clades clearly co-circulating in Uganda while no Africa 1C member was found. Moreover, sequences from Moyo and Ntoroko showed that both, the Africa 1A and 1B clades, circulate in dogs, while the Africa 1B only was isolated from dogs, jackals and fox of Jinja, Kabale, Kabarole, Namayingo and Kabongo districts, of the eastern, north eastern and western Regions of Uganda. Consequently, the presence of mixed lineages circulating between northern and western Uganda could be associated to the movement of dogs or wild animals like foxes, jackals, mongoose and hyenas along these regions (Lake Albert and the Semliki River). Moreover, the cross-border movements of animals between Sudan, Democratic Republic of Congo, Tanzania and Uganda could potentially contribute to the introduction of these two cosmopolitan main lineages in Uganda. Indeed, the location of Uganda at the crossroads of three major biogeographical regions (Afrotropical – Ethiopio-Somalian, Afrotropical – Sudano-Congolian, Afrotropical - Zambezian), may favor the local circulation of different rabies lineages from adjacent regions (Figs. 3, 4 and SM1).

The temporal phylogeography of rabies in Africa highlights 3 probable major origins of diversification of Africa 2, Africa 3 and Africa 4 clades from the West, South and North-East regions, respectively and estimates these events to initiate in a period ranging from 1800 to 1900 (Figs. 3, 4 and SM1, 2, 3 and 4). Moreover, the Africa 1 clade exhibits a phylogeographic structuration in 3 main clusters (1A: North-Eastern Africa to Uganda; 1B: Western to Central and South-Eastern Africa: 1B and 1C: East, mainly Madagascar), that suggests a gradual evolution of RABV lineage 1 in Africa from three major geographical origin (West, East and South) (Fig. 3, SM1, 2, 3 and 4). In addition, the clustering of few viruses (including 12RS266_9/Dog/2010/Moyo/Uganda reported by this study and U22488_Nigeria_RVU22488) in a rarely detected clade that early diverges from the root of Africa 1B clade, suggests the actual existence of yet unknown missing-link taxa and a continuous diversification of subclades (Fig. 3, SM1 and 2). This divergent clade, basal to Africa-1B, sometimes classified in a polyphyletic Africa 1B clade [25], or often omitted in more recent works, yet clearly forms a sister-clade to Africa 1B and, may be considered independently of Africa-1A and Africa-1B and seen as a separate lineage, that we propose to name Africa-1D here (Fig. SM2). This putative new 1D clade is based on: 1°) The well supported clustering of RABVs from Nigeria, Central African Republic, Uganda and Tanzania in a clade basal to Africa 1B; 2°) The genetic distance of these viruses to other Africa 1A and Africa 1B RABVs; 3°) The recent detection of RABVs in 2010 and 2011, from Uganda and Tanzania, respectively, which suggests the actual circulation of this clade in dogs or wildlife. These aspects strongly suggest the actual circulation of yet unknown RABVs from this proposed Africa 1D clade in the region.

Interestingly, even if some of these countries are distant, they all belong to the same biogeographical zone [17] (Fig. 3, SM1). At first, one can hypothesize that either 1/ a translocation of infected animals across the region may explain this pattern, 2/ a long time divergence with respect to the genetic distance between these viruses with West to East independent and gradual evolution, eventually occurring during several waves. However, given the paucity of the data that support these hypotheses, there is a great need for further study to detect other members of this sub-clade. Altogether, biogeography, long-distance and transborder movements are in favor of RABVs diffusion and a gradual long-term evolution of RABVs Africa 1 since or before the middle of the nineteenth century. The relaxed molecular clock analysis suggests that the diversification of this clade occurred at the latest during the second half of the twentieth century. Phylogenetic analyses link the circulation of one lineage 1B in Uganda (detected from 2010 to 2012 in all localities sampled) to Tanzanian and the episodic presence of a second lineage (in 2010 in Moyo), also to a Tanzanian lineage.

### Limitation of the study

The study had limited funding and time for the researcher to cover a wider scope of these two districts. The study design was retrospective and descriptive. The study implementation was focused on understanding the risk and identifying Rabies control and prevention gaps for the population. Also, the study sites where identified to assess the transborder risk of rabies circulation.

## Conclusions

This study demonstrated that rabies still remains a public health hazard in Uganda. Scarcity of post exposure prophylaxis services in the health centers was noted.

Low levels of knowledge regarding the importance of wound washing was observed during the study. People rarely knew the risk of encephalitis leading to death of the victim. There is a vital need to create and support programs aimed at rabies prevention and control in both schools and communities.

Dog vaccination at wide scale would probably lower the human cases and would stand for the next major step toward a successful prevention strategy. A strong collaboration between veterinary and medical sectors under a one health platform is also needed to ensure adequate availability of preventative services to the communities.

A bio surveillance (i.e. early warning of transborder risk) of the RABV or RABV like strains circulation among domestic and wild animals in Uganda is needed. Moyo and Ntoroko districts (north western & western Regions) had an active circulation of co-existing RABV African 1A and 1B clades in dogs. The districts of Jinja, Namayingo, Kabongo, Kabale and Kabarole (Southern, East and North-Eastern_Regions) presented a single African 1B clade only.

Despite the limited records available, our results suggest that the RABV diversity circulating in Africa is underestimated. We hypothesize that rabies strain diversification is still ongoing (this is supported by the emergence of a putative Africa 1D clade detected in Uganda) and favored by limited and imbalanced immunization coverage among animal populations, including unvaccinated dogs and other free-ranging domestic animals.

## Supplementary information

**Additional file 1: SM0.** Knowledge, Attitude and Practive survey questionnaire of Rabies in Uganda.

**Additional file 2: SM1.** Biogeography of rabies in Africa. Biogeographical zones (modified from Linder 2012 with kind permission of the authors) and countries of *Rabies virus* dispersion in sub-Saharan Africa with B) Locations of probable ancestral nodes.

**Additional file 3: SM2.** Rabies virus maximum likelihood phylogenetic tree in Africa. Main African rabies lineages with viruses detected following this work in Uganda.

**Additional file 4: SM3.** Temporal phylogeography leading to the actual distribution of *Rabies virus* in Africa. Time resolved phylogeographic diffusion of rabies in Africa according to a continuous model. Time-lapse (1875 to 2012) putative phylogeography leading to the actual distribution of rabies in Africa (Fig. 4).

**Additional file 5: SM4.** Putative phylogeographic temporal diffusion of *Rabies virus* in Africa over past 137 years. Animation of the probable dynamic of diversification of rabies major African clades during the 1875 to 2012 period of time.

## Data Availability

All genetic data are registered and accessible in Genbank, sequences have been deposited under IDs KJ133660 to KJ133687 (https://www.ncbi.nlm.nih.gov/genbank/). Abstraction format used in the study and dataset are available and accessible from the corresponding authors upon request at: omodomichael@gmail.com and meriadeg.legouil@normandie-univ.fr

## Abbreviations

DRC: Democratic Republic of Congo
DVO: District Veterinary Officer
MAAIF: Ministry of Agriculture, Animal Industry and Fisheries
MHCs: Medical Health Centers
MOH: Ministry of Health - Uganda
NADDEC: National Animal Disease Diagnostics and Epidemiology Centre
OIE: World organization of Animal Health
One Step RT-PCR: Reverse Transcription - Polymerization Chain Reaction
PEP: Post Exposure Prophylaxis
RABV: Rabies Virus
RSS: Republic of South Sudan
SPSS: Statistical Package for Social Sciences
μl: Microlitres

## References

[1] Fooks AR, Banyard AC, Horton DL, Johnson N, McElhinney LM, Jackson AC (2014). Current status of rabies and prospects for elimination. Lancet.

[2] Fooks AR, Cliquet F, Finke S, Freuling C, Hemachudha T, Mani RS, Muller T, Nadin-Davis S, Picard-Meyer E, Wilde H (2017). Rabies. Nat Rev Dis Primers.

[3] Paola B, De Battisti C, Dacheux L, Marciano S, Ormelli S, Salmoni A, Caennazo ST, Lepelltier A, Bourhy H, Capua I, Cattali G. Lyssa virus detection and typing using pyrosequencing. J Clin Microbiol. 2011:1932–8. 10.1128/JCM.02015-10 PMC3122702 21389152

[4] Sadeuh-Mba SA (2017). Molecular characterization and phylogenetic relatedness of dog-derived Rabies Viruses circulating in Cameroon between 2010 and 2016. PLoS Negl Trop Dis.

[5] Olarinmoye AO, Kamara V, Jomah ND, Olugasa BO, Ishola OO, Kamara A, Luka PD. Molecular detection of RABV strain with N-gene that clustered with China lineage 2 co-circulating with Africa lineages in Monrovia, Liberia: first reported case in Africa. Epidemiol Infect. 2018; cambridge.org/hyg. 10.1017/S0950268818003333 PMC6518606 30868993

[6] Troupin C, Dacheux L, Tanguy M, Sabeta C, Blanc H (2016). Large-scale Phylogenomic analysis reveals the complex evolutionary history of rabies virus in multiple carnivore hosts. PLoS Pathog.

[7] Ministry of Health, Uganda. Annual health sector performance Report. Financial Year 2015-2016 pdf.

[8] OIE (2013). Reference; Manual of Diagnostic Tests and Vaccines for Terrestrial Animals. Chapter 2.1.13. B.1.C.i.

[9] Lawrence Hamilton′s (2011). Statistics with Stata 10 sixth edition (updated Version).

[10] De Benedictis P, De Battisti C, Dacheux L, Marciano S, Ormelli S, Salomoni A, Caenazzo TS, Lepelletier A, Bourhy H, Capua I, Cattoli G (2011). Lyssavirus detection and typing using pyrosequencing. J Clin Microbiol.

[11] Katoh K, Standley DM (2013). MAFFT multiple sequence alignment software version 7: improvements in Performance and usability. Mol Biol Evol.

[12] Gouy M, Guindon S, Gascuel O (2010). SeaView version 4: A multiplatform graphical user Interface for sequence alignment and phylogenetic tree building. Mol Biol Evol.

[13] Drummond AJ, Rambaut A (2007). BEAST: Bayesian evolutionary analysis by sampling trees. BMC Evol Biol.

[14] Darriba D, Taboada GL, Doallo R, Posada D (2012). jModelTest 2: more models, new heuristics and parallel computing. Nat Methods.

[15] Drummond AJ, Ho SYW, Phillips MJ, Rambaut A (2006). Relaxed Phylogenetics and dating with confidence. PLoS Biol.

[16] Bielejec F (2016). SpreaD3: interactive visualisation of spatiotemporal history and trait evolutionary processes. Mol Biol Evol.

[17] Linder HP, De Klerk HM, Born J, Burgess ND, Fjeldså J, Rahbek C (2012). The partitioning of Africa: statistically defined biogeographical regions in sub-Saharan Africa. J Biogeogr.

[18] Sambo M, Lembo T, Cleaveland S, Ferguson HM, Sikana L, Simon C, Urassa H, Hampson K (2014). Knowledge, Attitudes and Practices (KAP) about Rabies Prevention and Control: A Community Survey in Tanzania.

[19] Wandeler AI, Matter HC, Kappeler A, Budde A (1993). 12 the ecology of dogs and canine rabies: A selective review. Revue Scientifique et technique (International Office of Epizootics).

[20] MAAIF - Ministry of Agriculture, Animal industry and Fisherie ( https://www.agriculture.go.ug/), 2011 report. Laboratory Diagnosis of Rabies in Jackal Samples Collected from Kidepo National Valley Game. Kabong District. Available on request.

[21] Meslin FX, Fishbein DB, Matter HC. Rationale and prospectus for rabies elimination in developing countries. Curr Topics Microbiol Immunol. 1994;187:1–26. 10.1007/978-3-642-78490-3_1 7859487

[22] Ozanne-Smith J, Ashby K, Stathakis VZ (2001). Dog bite and injury prevention – analysis, critical review, and research agenda. Injury Prev.

[23] Dietzschold B, Li J, Faber M (2008). Concepts in the pathogenesis of rabies. Future Virol.

[24] WHO. WHO expert consulatation on rabies. Second report. Geneva; 2012. https://who.int/rabies/resources/who_trs_982/en.

[25] Johnson N, McElhinney LM, Ali YH, Saeed IK, Fooks AR (2004). Molecular epidemiology of Canid rabies in Sudan: evidence for a common origin of rabies with Ethiopia. [research support, non-U.S. Gov’t]. Virus Res.

