# Supplementary figures and images for: Rabies in Uganda: rabies knowledge, attitude and practice and molecular characterization of circulating virus strains
Source: BMC Infect Dis. 2020 Mar 6;20:200. doi: 10.1186/s12879-020-4934-y (PMC7060555; doi:10.1186/s12879-020-4934-y)

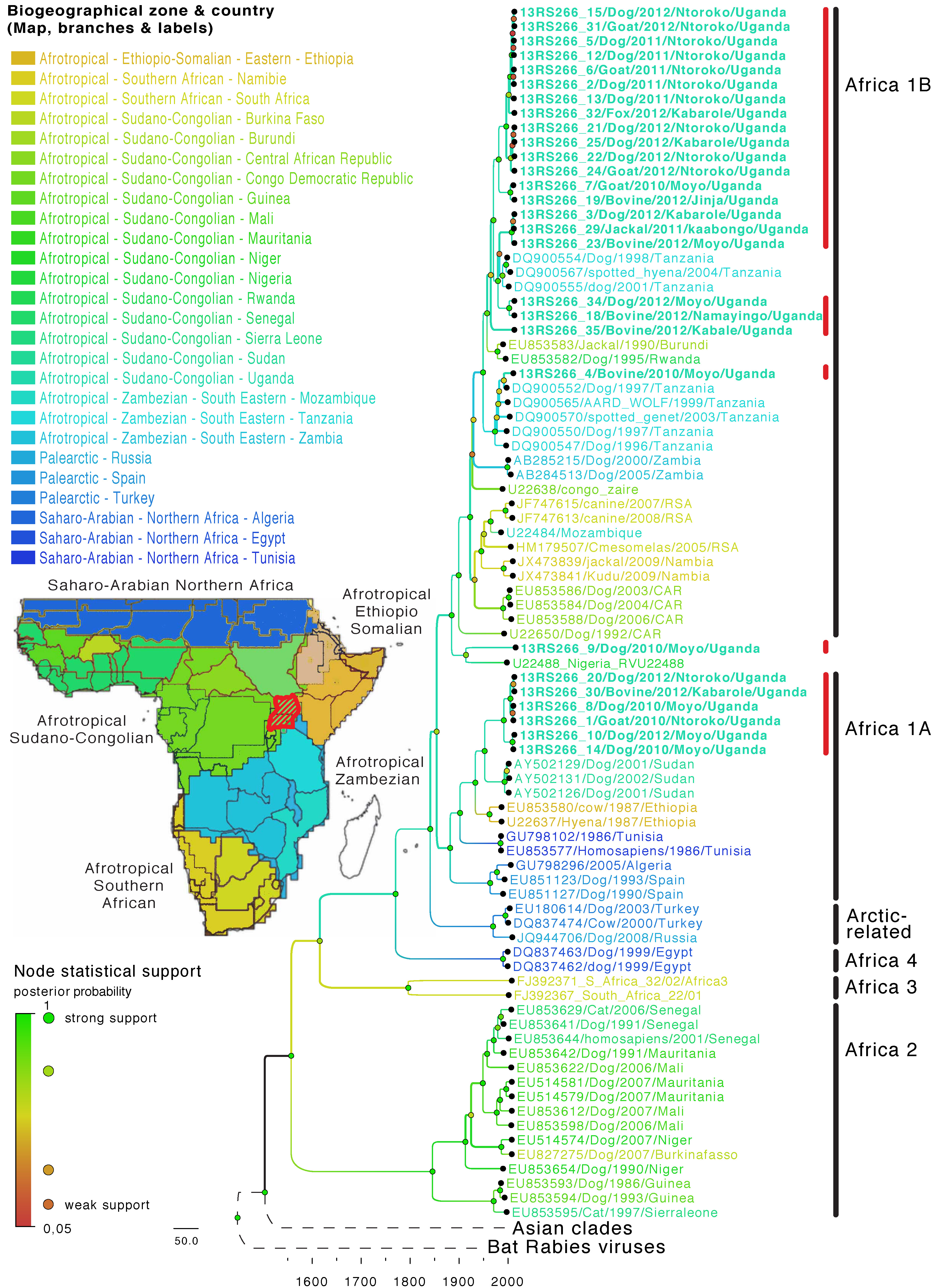

Supplement: Supplementary file 2 — Additional file 2: SM1. Biogeography of rabies in Africa. Biogeographical zones (modified from Linder 2012 with kind permission of the authors) and countries of Rabies virus dispersion in sub-Saharan Africa with B) Locations of probable ancestral nodes. [file 12879_2020_4934_MOESM2_ESM.jpeg]

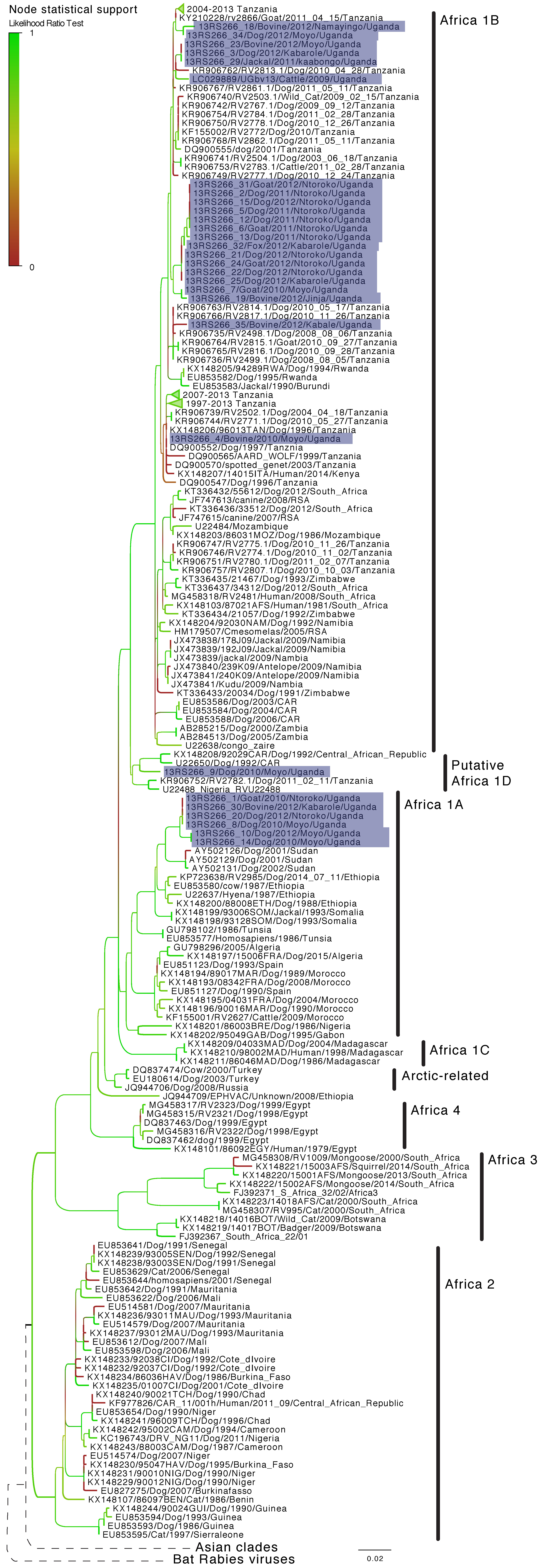

Supplement: Supplementary file 3 — Additional file 3: SM2. Rabies virus maximum likelihood phylogenetic tree in Africa. Main African rabies lineages with viruses detected following this work in Uganda. [file 12879_2020_4934_MOESM3_ESM.jpg]

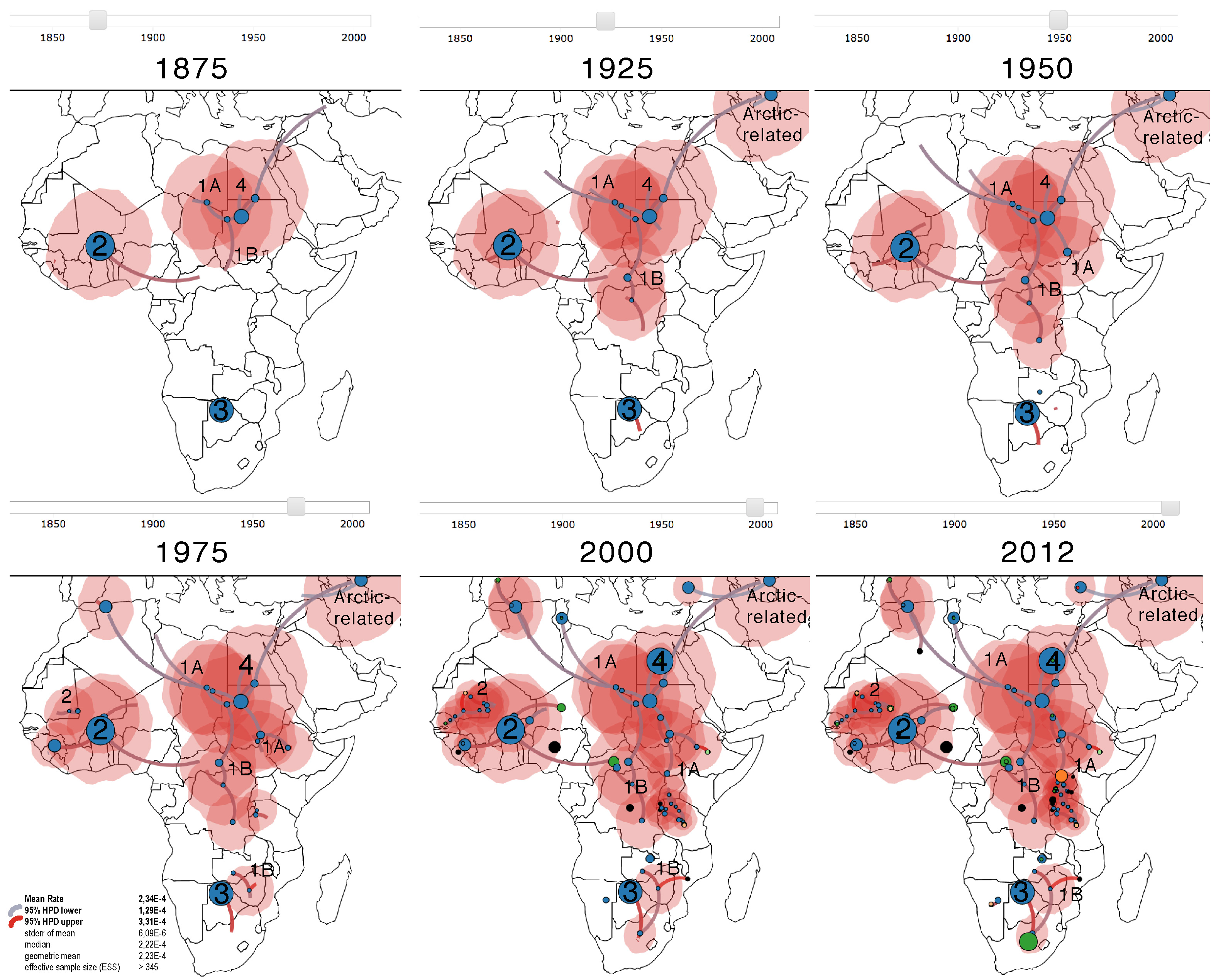

Supplement: Supplementary file 4 — Additional file 4: SM3. Temporal phylogeography leading to the actual distribution of Rabies virus in Africa. Time resolved phylogeographic diffusion of rabies in Africa according to a continuous model. Time-lapse (1875 to 2012) putative phylogeography leading to the actual distribution of rabies in Africa (Fig. 4). [file 12879_2020_4934_MOESM4_ESM.jpg]
