# Supplementary material for: Rabies in Uganda: rabies knowledge, attitude and practice and molecular characterization of circulating virus strains
Source: BMC Infect Dis. 2020 Mar 6;20:200. doi: 10.1186/s12879-020-4934-y (PMC7060555; doi:10.1186/s12879-020-4934-y)
Supplement: Supplementary file 3 [file 12879_2020_4934_MOESM1_ESM.odt]

Dear correspondents this exercise will be treated with confidentiality and your cooperation will be highly appreciated. 

Objective: To map the occurrence of rabies disease in Ntoroko and Moyo districts of Uganda 

Benefits: This study will generate comprehensive information about rabies occurrence in the selected districts and also the establish prevalent rabies virus strain in these districts.

 

Place…………………                                                                             Date…….………………

A)	Animal owner 

Name of the Dog owner/institution/community leader ………………………………..

Contact …………………………………………………………………………………

Age

 Sex of respondent:  Male                    Female

District ……………………………………………………………………………………………

Sub-county…………………………………………………………………………………..

Parish……………………………………………………………………………………

Village………………………………………………………………………………………

GPS…………………………………………………………………………………………

1)	Do you know rabies?

2)	What are the principal signs of rabies in dogs?

3)	Do you vaccinate your dogs or livestock for rabies?

4)	If yes, when have you carried out the last vaccination?

5)	How many a person did were bitten by rabies?

6)	Which kind of assistance would you provide to a person bitten by a dog?

8)	Which kind of assistance would you provide to a person bitten by a dog which is suspected to be rabies infected? 

9)	How many animals were affected by rabies in your households in the previous 2 years? (please specify the specie and number)

10)	Are you aware of the “World Rabies day” event? 

11)	 If yes, have you ever participated to such an event?

B)	District Veterinary officer

Name of the DVO ………………………………………………………………………

Contact ……………………………………………………………………………………

District ……………………………………………………………………………………

1)	What is the estimated dog population in your district?

2)	How many dogs and livestock (cattle) were vaccinated in 2009, 2010, 2011 and 2012? 

Table 1: showing species of animals vaccinated

3)	What is the current estimated stray dog population?

4)	How do you estimate it?

5)	Are there any attempts being implemented in the past 2 years to control the stray dogs population?

6)	If yes, please describe the methodology used.

7)	Please complete the following tables:

C)	Medical officer (at Rabies treatment center )

Name of the Medical Officer …………………………………………………………..

Contact …………………………………………………………………………………….

District ……………………………………………………………………………………………

Sub-county…………………………………………………………………………………..

Parish……………………………………………………………………………………

Village……

1)	Which is the first treatment given at presentation of a patient bitten by a rabies suspected dog?

2)	Do you have PEP available in this very moment?

3)	Do you have Rabies vaccines available in this very moment?

4)	Which PEP protocols and dosages do you apply?
